# Gender Sexuality Alliances and School Safety: Who Benefits Most, and Do Additive School-Led Practices Strengthen the Link?

**DOI:** 10.1007/s10964-024-01957-0

**Published:** 2024-02-28

**Authors:** T. M. L. Kaufman, W. J. Kiekens, L. Baams, H. M. W. Bos, M. E. De Looze

**Affiliations:** 1 Department of Pedagogy and Education, Utrecht University, Utrecht, Netherlands; 2 Department of Sociology/Interuniversity Center for Social Science Theory and Methodology, University of Groningen, Groningen, Netherlands; 3 Department of Pedagogy and Educational Sciences, University of Groningen, Groningen, Netherlands; 4 Research Institute of Child Development and Education, University of Amsterdam, Amsterdam, Netherlands; 5 Department of Interdisciplinary Social Sciences, Utrecht University, Groningen, Netherlands

**Keywords:** Gender and sexuality alliances, Sexual and gender diversity, School safety

## Abstract

While Gender and Sexuality Alliances (GSAs) are associated with higher acceptance of sexual diversity and lower bullying-victimization, it is unclear which individual and school-level attributes strengthen these associations. Nationally representative data (*N* = 1,567 students; *M*age = 15.4, *SD* = 0.16; 34% boys, 66% girls, 51% heterosexual, 49% sexually-diverse after propensity score matching) in 139 Dutch secondary schools were used. Multilevel regression analyses revealed that GSA presence was linked to more inclusive attitudes about sexual diversity and a safer disclosure climate among sexually-diverse students, and lower general bullying-victimization when the school had a GSA combined with school practices to tackle bullying. School professionals and researchers are recommended to recognize the significance of individual and school-level factors that affect GSA correlates.

## Introduction

Sexually-diverse students (i.e., students who experience attraction towards the same or multiple genders) consistently experience stigma, including bullying-victimization, and manifest health disparities in comparison to their heterosexual peers (e.g., Lucassen et al., 2017; Wittgens et al., 2022). This is particularly alarming during adolescence, a phase marked by heightened desires for peer group inclusion, where social exclusion or victimization exacts a significant social and emotional toll (e.g., McDougall & Vaillancourt, 2015). Moreover, adolescence is considered a critical period when exposure to stigma has a relatively substantial and long-term impact, because adolescents have not fully developed coping skills, self-efficacy and social power (Earnshaw et al., 2022). To support students, schools are increasingly encouraged, and in some jurisdictions mandated, to actively foster an inclusive school climate (Russell et al., 2021). An extensively studied approach to enhancing school safety is the establishment of a Gender and Sexuality Alliance (GSA). GSAs are student-initiated extracurricular school clubs designed for sexually-diverse and gender-diverse (SGD) students and their cisgender, heterosexual peers, providing social support and opportunities for school advocacy (Li et al., 2019). However, limited knowledge exists regarding individual attributes and school practices that determine who benefit the most from GSAs in terms of school safety (Poteat et al., 2017). This study explores potential variations in the associations between GSA presence and social safety 1) for sexually-diverse versus heterosexual students, and 2) for schools with and without additional school-led practices aimed at cultivating social safety.

### GSA Presence and Social Safety

In their pursuit of fostering an inclusive school climate, GSAs engage in a diverse array of activities, encompassing the provision of a platform for education and safety, leadership development, school-wide advocacy training, interpersonal support, and recreational pursuits (Poteat et al., 2017). Concrete illustrations of these activities include the display of posters challenging heterosexism, and the facilitation of training sessions for teachers focusing on homo- and transphobia within the school.

While GSAs primarily focus on SGD inclusion, their presence correlates with enhanced student well-being, school functioning, and a positive school climate for all students (e.g., Poteat et al., 2021). GSAs, thus, cultivate “social safety” at school reflected by both acceptance for sexually-diverse students and contributing to a generally improved social climate throughout the entire school marked by reduced levels of bullying-victimization (Russell et al., 2021). Moreover, it is important to note that GSA initiatives are designed to enhance the overall school climate, extending beyond the safety of GSA members alone. Substantiating this perspective, several prior studies have demonstrated that the presence of a GSA correlates with improved student outcomes and reduced bullying rates, irrespective of whether the students themselves were active GSA members (e.g., Ioverno et al., 2016).

### Do GSAs Benefit Sexually-Diverse and Heterosexual Students Equally?

While a GSA primarily focuses on sexual and gender diversity, it is also designed to benefit heterosexual students (e.g., Russell et al., 2021). All students stand to gain from a generally safer and more inclusive climate, and from reduced homophobic bullying—a phenomenon experienced not only by sexually-diverse but also by heterosexual students (Fish et al., 2023). However, the extent to which sexually-diverse and heterosexual students experience equal benefits remains uncertain (Poteat et al., 2017). Previous research has predominantly concentrated on sexually-diverse student samples initially targeted by GSAs, rather than considering the role GSAs might have for heterosexual students. This is crucial because it would offer insights into the role of a GSA for both the majority group within a school (i.e., heterosexual students) and the minority group that stands to benefit the most from having a GSA. One exception is a study using data from Californian schools, that showed no differences in strength of associations between SGD and heterosexual, cisgender students for bullying based on sexual attraction (Baams & Russell, 2021). Building upon this research, it is important to extend the inquiry to encompass both sexual diversity-specific and overall social safety as targeted by a GSA. Additionally, incorporating a statistical correction for the varied sample sizes within these groups during comparative analyses would enhance the robustness of such comparisons between sexually-diverse and heterosexual students. Last, it is important to expand upon the existing knowledge within the Dutch context. This is considered a relevant context: While Dutch schools are legally required to foster an inclusive school climate (Opstelten, 2012), sexually-diverse students still face negative attitudes, discrimination and bullying at school (Kaufman & Baams, 2021).

Theoretically, divergent expectations can be postulated concerning distinct associations between GSA presence and social safety for heterosexual and sexually-diverse students. Drawing upon person-environment fit theory (Calzo et al., 2020) the impact of a school context on an individual student depends on the alignment between the context and the student’s needs. Intuitively, it may be posited that a GSA in schools offers most social safety for sexually-diverse students, given their heightened need for reduced stigma and for acceptance. Moreover, a GSA provides validation for sexual minority identities, which may contribute to diminishing sexually-diverse student’s internalized homophobia, an issue less prevalent among heterosexual students. Also, a GSA could represent the sole safe space for sexually-diverse students to discuss their sexual orientation or seek refuge from victimization, whereas heterosexual students may find support for socially challenging situations in spaces outside of the GSA. To this end, a GSA primarily addresses issues central to the lives of sexually-diverse students, such as feeling accepted, providing a safe space for disclosing diverse sexual orientations, or for mitigating bullying-victimization.

Alternatively, and somewhat counterintuitively, an opposing expectation emerges that heterosexual students might experience greater social safety in the presence of a GSA compared to their sexually-diverse peers. Heterosexual students generally report to feel socially safer than their sexually diverse peers. GSA’s positive associations with more accepting norms regarding sexual diversity might also benefit heterosexual students, further increasing their feelings of social safely. Sexually diverse students also benefit from these norms, but do not reach the higher levels of heterosexual students as sexually-diverse students often harbor ingrained chronic vigilance towards stigmas even when GSA’s are present. Trusting the school to be a “safe space” when a GSA is present may, thus, be more challenging for sexually-diverse students.

Moreover, heterosexual students may experience greater mean-level GSA benefits compared to sexually-diverse peers: The latter group likely consists of more individuals who are severely disappointed when the GSA fails to meet their needs, who weaken the average association between social safety and GSA presence. A sexually-diverse student may encounter intensified feelings of marginalization when the highly needed GSA does not improve their situation, a phenomenon acknowledged as the “healthy context paradox” –i.e., feeling worse in a context where you are one of the only ones who is not helped (Kaufman et al., 2023).

### Can School-Led Practices Strengthen the Positive Correlates of Student-Led GSAs?

In addition to potential variations in GSA correlates tied to student attributes like sexual orientation, the associations between GSA presence and social safety may also differ depending on school-level factors. From a socioecological standpoint, it is argued that a stand-alone GSA, led by students, may not be adequate to cultivate an inclusive school climate; a supportive school context is deemed a necessary foundation for such initiatives (Calzo et al., 2020). Particularly during adolescence, when resisting peer pressure is challenging and the desire for peer group inclusion is heightened, adults at school play a crucial role in regulating challenging social behaviors and contributing to safe spaces (Espelage, 2014). In this regard, adult school staff serves as important source of support for students who face peer victimization or encounter stigma (McCauley et al., 2023).

In the specific context of GSAs, both queer pedagogy theory (Lapointe, 2015) and the program theory of GSAs (Schlief et al., 2023) align with this viewpoint by positing that a GSA cannot effectively benefit students if it operates as a separate, isolated, and marginal club within a heteronormative school context. Instead, the institution itself should actively incorporate supportive messages into daily learning practices and the foundation of the school culture. An optimal approach, thus, involves complementing the student-initiated GSA by school-led practices promoting social safety (Schlief et al., 2023).

Examples of such practices include concrete activities undertaken by the school to cultivate safe climates, such as organizing extracurricular activities, theme days or projects, or parent meetings focusing on acceptance of sexual and gender diversity (SGD-specific practices), which have been related to social safety. For instance, attending a school with positive representations of LGBTQ+ topics in the curriculum or having supportive educators relates to fewer experiences of homophobic bullying among sexually-diverse students (Kosciw et al., 2021). In school contexts with such inclusive practices, it is more likely that the school culture further validates and supports the student-initiated GSA activities.

Further, school practices not specifically centered around sexual orientation but aimed at addressing general bullying may also nurture a safe environment for a GSA. Generally, anti-bullying strategies impart prosocial methods to students, guiding them in establishing a safe group atmosphere and responding effectively when witnessing peers facing bullying or stigma (Salmivalli et al., 2021). This prosocial environment may serve as a safe space for a GSA to discuss sensitive topics, and may contribute to the long-term sustainability of the GSA (Poteat et al., 2017). Furthermore, when the school, not just the GSA, takes a unequivocal stance against bullying, it likely becomes easier for GSA members to garner support from school staff when confronted with intimidating situations (Watson et al., 2010).

Despite the acknowledged importance of the school context for a GSA, few studies have explored the additional role of school practices in relation to social safety. Two studies suggest that inclusive school policies—i.e., those designed to protect sexually-diverse students—can enhance the social support provided by GSAs. In one study, the presence of a GSA was only associated with reduced homophobic and gender-based bullying, as well as increased general support from classmates and teachers when coupled with SGD-focused school policies (Day et al., 2020). In another study, the combination of GSA presence and anti-homophobic bullying school policies was found to contribute to the reduction of sexual-orientation discrimination (Saewyc et al., 2014). These findings support the expectation that the combination of a student-initiated GSA and an inclusive school context benefits sexually-diverse students. Nevertheless, school policies do not always align seamlessly with actual practices. It is, therefore, important to study to what extent prevailing practices—whether specifically addressing sexuality or oriented towards general bullying—correlate with the associations between GSA presence and school safety.

## Current Study

Despite the existing body of research on the associations between GSA presence and social safety, understanding how this link varies among individuals and school contexts remains unclear. The first aim of this study was to investigate whether the association between the presence of a GSA and social safety of students differed for sexually-diverse and heterosexual students. The second aim was to assess whether the association between the presence of a GSA and social safety of students was affected by the presence of existing school practices (e.g., extracurricular projects, theme days or parent meetings) designed to foster acceptance of sexual diversity or to enhance general social safety. To address these aims, this study explored potential differences between students in schools with and without a GSA, focusing on their individual perceptions of social safety. In this paper, social safety encompassed both safety specific to sexual diversity and general social safety. Sexual diversity-specific safety included the extent to which students felt safe being open about their sexual attraction at school and the inclusivity of their own attitudes toward sexual diversity. Additionally, general social safety was reflected by experienced levels of bullying-victimization. Drawing upon person-environment fit theory, it was hypothesized that GSA presence would be related to more social safety, and that this association was different between sexually-diverse and heterosexual students. The anticipated directions and effects were exploratory, given the mixed theoretical expectations and lack of prior research. Moreover, informed by socioecological theories that emphasize the importance of the broader school context of GSAs, it was hypothesized that school-led practices aimed at fostering social safety would enhance the associations between GSA presence and social safety.

## Methods

### Procedures and Sample

Two measurement waves (2017, 2021) of the nationally representative Dutch Health and Behaviour in School-Aged Children (HBSC) study were used to have sufficient analytical power (Moor et al., 2020), using a repeated cross-sectional design. The sampling and survey procedures for the different measurement waves were identical. The study included data from 156 schools (including 87 schools from the 2017 cohort, and 69 schools from the 2021 cohort) that provided general secondary education, and 13,125 students aged 11 to 16 (*M*age = 14.1, *SD* = 1.64) attending these schools. Students attended in-person classes during both measurement periods, as Covid-19 measurements transitioning to online education had concluded in January 2021, and the 2021 data collection started in Fall. The samples were obtained using a two-stage random sampling procedure. First, schools were stratified and drawn proportionally according to the level of urbanization, based on a national file of secondary schools, provided by the ministry of Education, Culture and Science. Second, within each school two to five classes (depending on school size) were selected randomly from a list of all classes provided by each participating school. Within the selected classes, all students were drawn as a single cluster. Data were collected via web-based questionnaires.

Staff and student surveys were completed for each school. One staff member per school filled in several questions about GSA presence and school-led practices that the school had in the past year to foster social safety. This staff member was typically the counsellor (34.0%), the school principal (25.1%), or the department chair (20.4%). In addition to school staff, students completed a survey about their social experiences, attitudes, and perceived school climate. Participants were assured of the anonymity and confidentiality of their responses. Participant non-response rates were low (<10%) and mainly because of illness.

Of the total sample, *N* = 17 schools and *N* = 1,294 students did not report information about GSA presence, resulting in a sample of *N* = 139 schools and *N* = 11,831 students. Most students were heterosexual (87.0%) and others were sexually-diverse (2.0% same-gender/sex attracted, 4.0% both-gender/sex attracted, 6.9% unsure).

To address the limitations with different sample sizes for heterosexual and sexually-diverse groups, and to account for potential confounding factors in the examined associations, this study followed previous studies on sexually-diverse and heterosexual students by implementing propensity score matching procedures (Martin-Storey & Fish, 2018). These procedures rendered the heterosexual and sexually-diverse student comparison groups as similar as possible via matching on demographic variables. First, heterosexual and sexually-diverse students were compared on the demographic covariates that were also used in the main analyses (sex, migration background, family affluence, cohort, age) while correcting for nesting in schools. As presented in Table 1, proportion scores and mean scores were significantly different (i.e., non-overlapping confidence intervals) on nearly all factors based on sexual attraction status. Thus, this study performed propensity score matching to derive a matched comparison sample of sexually-diverse students who did not statistically differ from their heterosexual peers. The default caliper point difference of 0.02 was selected to maximize the similarity between sexually-diverse and heterosexual groups and to minimize the loss of participants for failure to match (Morgan & Harding, 2006). Post-hoc comparative analyses revealed no significant differences between sexually-diverse students and the propensity-matched heterosexual students, as indicated in Table 1. Additionally, disparities in propensity scores between the groups were notably reduced when comparing the matched and non-matched samples. Consequently, the subsequent analyses were performed using the sexually-diverse and propensity-matched sample.

**Table 1:** Differences across sexual attraction prior and following propensity score matching, adjusted for nesting in schools

	Differences across sexual attraction status prior to propensity score matching				Differences across sexual attraction status following propensity score matching			
	Heterosexual	95% CI	Sexually-diverse.	95% CI	Heterosexual	95% CI	Sexually-diverse	95% CI
Sex (girl)	48.4	46.7; 50.1	66.1*	63.0; 69.1	66.1	53.7; 76.7	66.1	62.9; 69.2
Migration background	21.6	18.9; 24.7	23.4	20.4; 26.8	23.3	19.9; 32.7	23.2	20.4; 26.2
Family affluence								
Low	0.08	0.07; 0.09	0.10*	0.08; 11.8	0.10	0.06; 0.15	0.10	0.08; 0.12
Moderate	37.8	36.3; 39.1	41.0*	38.2; 43.8	0.41	0.30; 0.53	0.41	0.48; 0.44
High	54.7	52.7; 56.8	49.0*	46.1; 52.0	0.49	0.37; 0.61	0.49	0.46; 0.52
Cohort ‘21	41.1	33.2; 49.3	54.8*	45.8; 63.6	0.55	39.2; 69.3	0.55	0.46; 0.64
Age	14.2	14.1; 14.3	13.9*	13.8; 14.1	13.9	13.6; 14.3	13.9	13.8; 14.1

Variables were compared based on proportion scores, except for the linear variable age, for which mean scores were compared

*significant difference (*p* < 0.05)

As shown in Table 2, the final sample included *N* = 1,567 students (51.1% heterosexual, 48.8% sexually-diverse; *M*age = 15.4, *SD* = 0.16), of which about two-thirds were girls (65.5%) and 77.6% identified as Dutch (non-migration background). Students who reported a migration background were Moroccan (3.9%), Turkish (1.9%), Surinamese (2.4%), German (1.2%), Antillean (1.3%) or had another background (11.7%). Most students reported a high (50.6%) or moderate (39.3%) family affluence, and only a small number reported a low family affluence (10.1%). In terms of school characteristics, 37.4% of schools reported to have a GSA. About half of the schools had, in the past year, undertaken SGD-specific school-led practices (51%), and/or general anti-bullying practices (71%).

**Table 2:** Sample descriptives: percentages adjusted for nesting in schools

	Adj. %	95% CI
% students		
Sexually-diverse^a^		
Heterosexual	51%	43; 59
Sexually-diverse	49%	41; 57
Sex/Gender		
Girl	34%	28; 41
Boy	66%	59; 72
Migration background^b^		
No	78%	72; 82
Yes	22%	18; 28
Family affluence		
Low	10%	8; 13
Moderate	39%	33; 46
High	51%	43; 58
% schools		
GSA presence		
No	59%	51; 67
Yes	35%	28; 44
Maybe	5%	3; 11
School-led SGD-specific practices^c^		
No	49%	41; 57
Yes	51%	43; 59
School-led anti-bullying practices		
No	29%	22; 36
Yes	71%	64; 78
Cohort		
2017	56%	54; 58
2021	44%	36; 52

^a^Self-reported sexual attraction to same and/or opposite gender, same or both-gender attracted, or who were unsure, were coded as sexually-diverse

^b^Non-Dutch migration background

^c^School has actively addressed the topic of SGD-inclusion in the past year

### Instruments

#### School staff survey

##### GSA presence

The presence of a GSA was assessed by asking school staff about GSA presence in their school with the question “Does your school have a GSA? A GSA is a group of students (gay, straight, transgender; often supported by a teacher) that enables everyone to be themselves at school, regardless of sexuality or gender” (0 = no, 1 = yes). The third answer option “I don’t know” (checked by 5.8% [N = 8] of the schools) was coded as missing in the main analyses, and only used in sensitivity analyses.

##### School practices

School-led practices to foster social safety were assessed with a question that school staff answered about the presence of school-led practices: “Which subjects did the school actively address last year in the form of extracurricular practices, theme days or projects, or parent meetings?” Participants could check the boxes for “Being gay, transgender students” (*SGD-specific practices*) and/or for “Bullying at school” (*general anti-bullying practices*) when applicable. Both were used as separate variables (0 = no, 1 = yes).

#### Student survey

##### Sexual diversity-inclusive attitudes

Students’ attitudes towards sexual diversity were assessed with three questions: “Gay boys and lesbian girls can be my friends”, “I find it disgusting when two boys kiss”, and “I find it disgusting when two girls kiss”. Answer options ranged from 1 = completely agree to 5 = completely disagree (first item was reverse-coded). The sixth answer option “I have never considered this” (9%) was coded as missing. A mean score was created based on the three items, *r*: = −0.37 (items 1 and 3), *r* = −0.53 (items 1 and 2) to *r* = 0.75 (items 2 and 3), that formed a reliable scale (Cronbach’s alpha = 0.79). Higher scores reflected more accepting attitudes. Analyses that used this measure controlled for participants’ answer on a fourth question (i.e., used as covariate): “I find it disgusting when a boy and a girl kiss” (same answer options). These questions stem from several Dutch studies on attitudes towards sexually-diverse people (Roos et al., 2014).

##### Safe disclosure climate

Students’ perception of the extent to which it was safe to disclose one’s diverse sexual orientation was assessed with the question ‘When one of your schoolmates would be gay, do you think that they could honestly tell this to others at school?” (0 = no, 1 = yes, only to their friends, 2 = yes, to everyone at school). The fourth answer option “I don’t know” was coded as missing.

##### Bullying-victimization

Two complementary measures were used to assess bullying-victimization. First, bullying-victimization frequency was assessed with the bullying item from the Olweus (1993) survey. Participants were first presented with the definition of bullying: “Someone is bullied when someone else, or a group of others, regularly says or does negative, hurtful things towards them. It is also considered bullying when someone is teased in a way that they do not like or is purposely excluded. The person who bullies has more power than the person being bullied and wants to harm or hurt the person. It is NOT bullying when two people of the same strength or size are arguing or fighting.” The definition was followed by the question: “How often have you been bullied at school in the last few months?” (0 = never, to 4 = multiple times per week).

As a robustness check, an additional measure was included: global bullying-victimization, which did not specifically assess the frequency of such experiences. Students responded the extent to which they agreed with the statement “I am bullied” (0 = not true, 1 = somewhat true, 2 = completely true) from the Strengths and Difficulties Questionnaire (Goodman, 1997).

##### Sexual attraction

Students’ sexual attraction was assessed with the question “Are you attracted to boys, girls, or both?” Participants could check one of the options (boys, girls, both, I don’t know) and were coded as heterosexual (0) when they checked the opposite gender. Those who checked the same gender, both, or who were unsure were coded as sexually-diverse (1). This measure is commonly used to assess sexual attraction and orientation among adolescents (Baams & Kaufman, 2023).

##### Demographic covariates

Participants reported their own and parents’ country of birth, and the subcategories were collapsed into two categories (0 = no migration background, 1 = migration background). Family affluence was assessed using the Family Affluence Scale (Currie et al., 1997). Participants were asked to answer six questions on affluence in their family (e.g., “Does your family own a car, van, or truck?”). A sum score reflecting family affluence was calculated and recoded as 0 = low, 1 = middle, and 2 = high, based on HBSC scoring procedures (Torsheim et al., 2016). Participants also reported their gender/sex (“Are you a boy or a girl?” 0 = boy, 1 = girl) and their birth date, based on which their age was calculated.

### Analytic Strategy

Multilevel regression analyses clustered at the school level were conducted in Stata 16.0. Student-level attributes were estimated at the within-person level, and school practices were estimated at the between-person level. Associations between GSA presence and social safety outcomes, and interaction effects (cross-level interaction GSA presence x sexually-diverse, between-level interactions GSA presence x school-led sexual diversity-inclusion practices, GSA presence x school-led anti-bullying practices) were estimated for every outcome separately (sexual diversity-inclusive attitudes, safe disclosure climate, two indicators of bullying-victimization). The model, thus, included both fixed effects and random effects across schools.

Two different indicators of bullying-victimization were used because although the first measure is considered the “golden standard”, it has been criticized because it focuses on frequency and ignores that incidental experiences of bullying-victimization can also be harmful (Volk et al., 2014). To maximize the robustness of the analyses, the measure was complemented with an alternative measure that focused on students’ general perception of whether they were bullied (“global bullying-victimization”), which showed a large, albeit not perfect, overlap (*r* = 0.60).

Predictive margins were estimated when a significant interaction effect was found, which corrected for the multilevel structure in the same way as the analyses did. Standardized effects were not available for multilevel analyses but explained variance on both levels (individual, school) were calculated for every model.

Additional analyses tested the main associations between being *unsure* whether the school had a GSA versus reporting to have no GSA. Given the small number of schools reporting to be unsure of having a GSA (*n* = 8), moderation effects were not additionally tested within this sample.

## Results

First, intercorrelations were examined at the school level and at the student level, separately. School-level intercorrelations showed that schools with a GSA were slightly more likely to also have SGD-specific practices (*r* = 0.17, *p* = 0.049) but not general anti-bullying practices (*r* = −0.05, *p* =0.577), than schools without a GSA. Further, schools that reported SGD-specific practices were also moderately more likely to have anti-bullying practices than schools without SGD-specific practices (*r* = 0.31, *p* < 0.001).

Intercorrelations at the student level were analyzed independently for sexually-diverse and heterosexual students, but appeared to be similar between these groups. As presented in Table 3, the two different victimization measures based on frequency and global presence were largely related to each other. Further, this table presents the small positive associations between more sexual diversity-inclusive attitudes and safer disclosure climate, and between safer disclosure climate and lower levels of bullying-victimization across both measures. Sexual diversity-inclusive attitudes and bullying-victimization across both measures were unrelated.

**Table 3:** Correlations between within-person key variables across heterosexual (below diagonal) and sexually-diverse (above diagonal) students

	1.	2.	3.	4.
1. Sexual diversity-inclusive attitudes	**–**	**0.29**	−0.03	−0.03
2. Safe disclosure climate	**0.23**	–	**−0.16**	**−0.13**
3. Victimization (frequency)	0.01	**−0.10**	–	**0.61**
4. Victimization (global presence)	−0.01	**−0.10**	**0.54**	–

Bold values represent significant correlations (*p*s <0.001)

### Multilevel Linear Regression Analyses: GSA Presence and School Safety

The results of the multilevel linear regression analyses, presented in Table 4 (model 1), showed that GSA presence was significantly associated with the sexuality-specific but not general social safety indicators: Students with a GSA in their school, compared to those without one, reported more sexual diversity-inclusive attitudes, more perceived safety to disclose one’s diverse sexual orientation at school, but no differences in bullying-victimization across both measures. Effects in terms of pseudo-*R*^2^ were small to moderate.

**Table 4:** Linear multilevel regression: social safety across combinations of GSA presence and school practices

	Sexual diversity-inclusive attitudes				Safe disclosure climate				Bullying-victimization (frequency)				Bullying-victimization (global presence)			
	*b*	95% CI	pseudo-*R*^*2*^		*b*	95% CI	pseudo-*R*^*2*^		*b*	95% CI	pseudo-*R*^*2*^		*b*	95% CI	pseudo-*R*^*2*^	
			ind.	school			ind.	school			ind.	school			ind.	school
Model 1. GSA main effect			0.47	0.05			0.07	0.34			0.01	0.04			0.02	0.09
GSA	**0.16**	0.01; 0.31			**0.13**	0.01; 0.25			−0.07	−0.18; 0.04			−0.03	−0.11; 0.06		
Sexually-diverse	**0.67**	0.50; 0.84			**0.03**	−0.12; 0.19			**0.32**	0.14; 0.51			0.19	0.10; 0.28		
SGD-specific practices	−0.02	−0.16; 0.13			0.08	−0.03; 0.20			−0.07	−0.17; 0.04			−0.02	−0.09; 0.06		
Anti-bullying practices	−0.12	−0.27; 0.02			−0.04	−0.16; 0.08			0.11	−0.00; 0.22			0.02	−0.06; 0.10		
Model 2. GSA x sexually-diverse			0.48	0.06, 0.14^a^			0.06	0.43, 0.04			0.02	0.03, 0.01			0.02	0.06, 0.01
GSA	−0.14	−0.43; 0.14			−0.21	−0.51; 0.09			−0.08	−0.20; 0.05			**0.01**	−0.15; 0.18		
Sexually-diverse	**0.48**	0.25; 0.71			−0.12	−0.37; 0.13			0.29	0.14; 0.44			**0.22**	0.09; 0.36		
SGD-specific practices	−0.02	−0.16; 0.13			0.01	−0.14; 0.17			−0.08	−0.17; 0.02			−0.02	−0.09; 0.06		
Anti-bullying practices	−0.13	−0.27; 0.02			−0.00	−0.15; 0.14			**0.13**	0.04; 0.21			0.02	−0.06; 0.10		
GSA x sexually-diverse	**0.34**	0.19; 1.10			**0.38**	0.08; 0.68			0.02	−0.15; 0.19			−0.07	−0.25; 0.11		
Model 3. GSA x SGD-specific practices			0.48	0.11			0.07	0.47			0.01	0.06			0.02	0.16
GSA	**0.12**	−0.08; 0.31			**0.10**	0.13; 0.40			−0.01	−0.14; 0.11			0.07	−0.06; 0.21		
Sexually-diverse	**0.67**	0.50; 0.84			0.07	−0.00; 0.11			**0.30**	0.21; 0.39			**0.19**	0.10; 0.28		
SGD-specific practices	0.06	−0.08; 0.19			0.06	−0.02; 0.18			−0.05	−0.16; 0.07			0.03	−0.05; 0.12		
Anti-bullying practices	−0.13	−0.27; 0.02			0.02	−0.08; 0.10			**0.13**	0.05; 0.08			0.03	−0.05; 0.11		
GSA x SGD-specific practices	0.09	−0.18; 0.36			−0.09	−0.37; 0.18			−0.09	−0.24; 0.07			−**0.17**	−0.32; −0.01		
Model 4. GSA x anti-bullying practices			0.48	0.09			0.07	0.55			0.01	0.06			0.02	0.10
GSA	**0.07**	−0.11; 0.25			0.00	−0.25; 0.25			0.05	−0.09; 0.19			0.15	−0.03; 0.33		
Sexually-diverse	**0.67**	0.51; 0.84			0.07	−0.11; 0.26			**0.30**	0.15; 0.26			**0.19**	0.10; 0.28		
SGD-specific practices	−0.03	−0.17; 0.11			0.02	−0.13; 0.18			−0.07	−0.17; 0.02			−0.01	−0.07; 0.08		
Anti-bullying practices	−0.21	−0.39; 0.02			−0.02	−0.21; 0.18			**0.19**	0.08; 0.29			**0.10**	0.02; 0.18		
GSA x anti-bullying practices	0.20	−0.07; 0.47			0.08	−0.21; 0.36			−**0.17**	−0.33; −0.01			−**0.31**	0.42; −0.05		

Covariates were age, gender, sexual diversity, migration background, family welfare, cohort, school-led SGD-specific practices and anti-bullying practices. Bold values represent significant effects

*Ind* individual-level, *school* school-level

^a^Two pseudo-*R*^2^ values were provided for models that included a random slope interaction effect (level-1 sexually-diverse x level-2 GSA): the first value reflects variance explained by level-2 variables, the second value reflects variance explained by the random slope interaction sexually-diverse x GSA. For models 3 and 4, explained variance of the interaction effect was reflected by the difference between explained variance of the direct effects model by level-2 predictors versus the explained variance of the model with interaction effect by level-2 predictors

Next, mixed moderation analyses tested whether the strength of the associations between GSA presence and the four social safety indicators differed depending on student’s sexual attraction or the presence of school-led practices to foster social safety. The results are discussed by indicator of social safety (see Table 4).

**Fig. 1:**
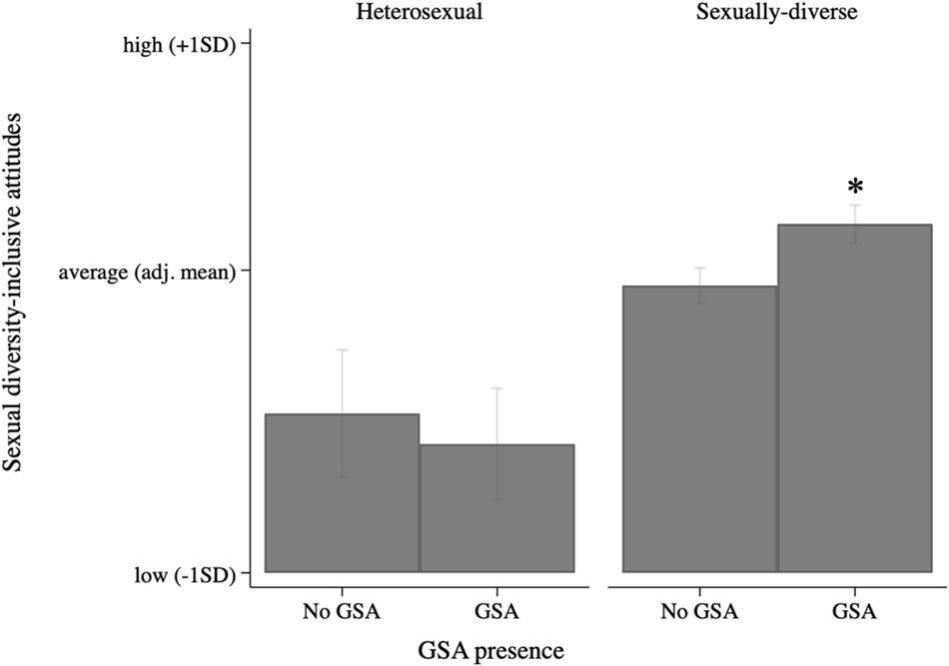
Interaction of GSA presence and sexual attraction on sexual diversity-inclusive attitudes. Note. *significant difference (*p* < 0.05) between no GSA versus GSA. Estimated predictive margins: for heterosexual students, no GSA (Margins = 3.62, 95%CI [3.39; 3.86]) versus GSA present (Margins = 3.51, 95%CI [3.31; 3.72]; for sexually-diverse students, no GSA (Margins = 4.10, 95%CI [4.03; 4.17]) versus GSA present (Margins = 4.33, 95%CI [4.26; 4.40])

For sexual diversity-inclusive attitudes, a significant interaction effect between GSA presence and sexual attraction (model 2) was found that explained 14% of the variance in sexual diversity-inclusive attitudes. Only for sexually-diverse students, attending a school with a GSA was related to significantly more inclusive attitudes towards sexual diversity than attending a school without a GSA, as shown in Fig. 1. Associations between GSA presence and sexual diversity-inclusive attitudes were not moderated by the presence of either school-led SGD-specific (model 3) or anti-bullying practices (model 4).

**Fig. 2:**
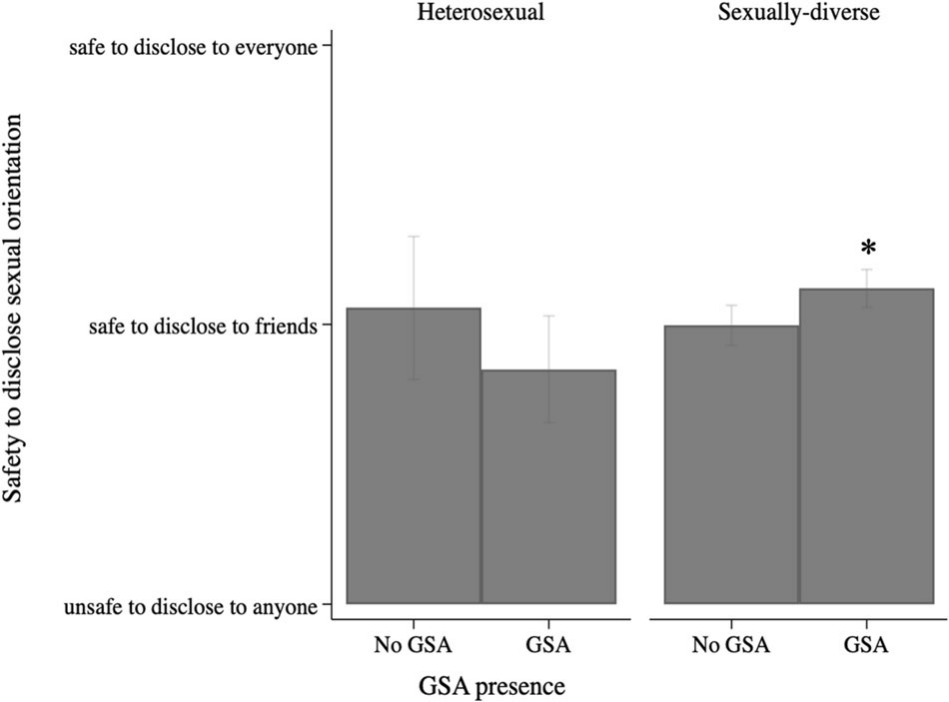
Interaction of GSA presence and sexual attraction on safe disclosure climate. Note. *significant difference (*p* < 0.05) between no GSA versus GSA. Estimated predictive margins: for heterosexual students, no GSA (Margins = 1.06, 95%CI [0.80; 1.32]) versus GSA present (Margins = 0.84, 95%CI [0.65; 1.03]); for sexually-diverse students, no GSA (Margins = 1.00, 95%CI [0.93; 1.07]) versus GSA present (Margins = 1.13, 95%CI [1.06; 1.20])

Regarding safe disclosure climate, again a significant interaction effect between GSA presence and sexual attraction (model 2) was found that explained 4% of the variance in safe disclosure climate. Only for sexually-diverse students, being in a school with a GSA was linked with more perceived safety to disclose one’s diverse sexual orientation, as shown in Fig. 2. Associations between GSA presence and safe disclosure climate were not moderated by the presence of either school-led SGD-specific (model 3) or anti-bullying practices (model 4).

**Fig. 3:**
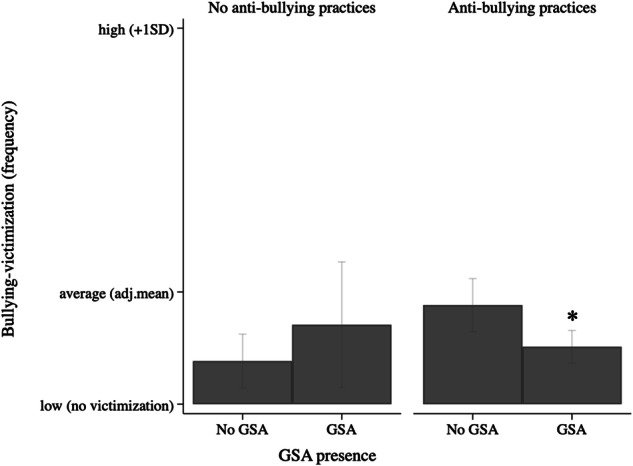
Interaction of GSA presence and anti-bullying practices on bullying-victimization frequency. Note. *significant difference (*p* < 0.05) between no GSA versus GSA. Estimated predictive margins: for no anti-bullying practices, no GSA (Margins = 1.16, 95%CI [1.06; 2.22]) versus GSA present (Margins = 1.29, 95%CI [1.12; 1.46]; for anti bullying practices present, no GSA (Margins = 1.24, 95%CI [1.17; 1.31]) versus GSA present (Margins = 1.15, 95%CI [1.10; 1.20]

For bullying-victimization frequency, a significant interaction effect was found between GSA presence and school-led anti-bullying practices (model 4), that explained 2% of the variance in bullying-victimization. Students in schools with anti-bullying practices reported significantly less frequent bullying-victimization when they also had a GSA, while for students in schools that did not actively lead anti-bullying practices, having a GSA at school was not related to bullying-victimization, see Fig. 3. Thus, only the combination of school-led anti-bullying practices and GSA presence was associated with less frequent bullying-victimization levels. There were no moderation effects of sexual attraction (model 2) or school-led SGD-specific practices (model 3).

**Fig. 4:**
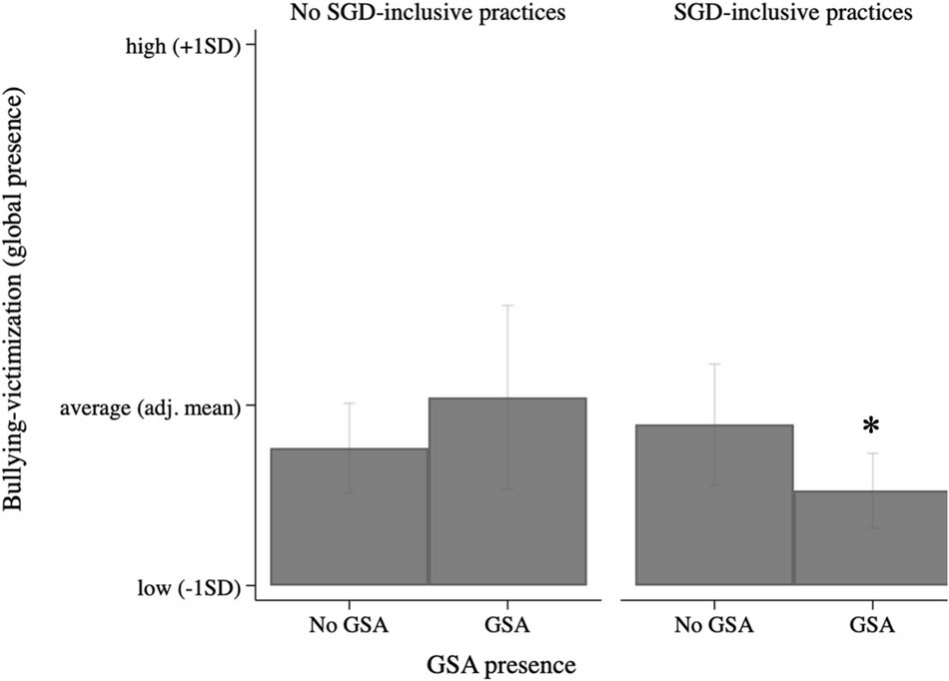
Interaction of GSA presence and SGD-specific practices on bullying-victimization (global presence). Note. *significant difference (*p* < 0.05) between no GSA versus GSA. Estimated predictive margins: for no SGD-specific practices, no GSA (Margins = 1.20, 95%CI [1.13; 1.26]) versus GSA present (Margins = 1.27, 95%CI [1.14; 1.40]; for SGD-specific practices present, no GSA (Margins = 1.23, 95%CI [1.15; 1.32]) versus GSA present (Margins = 1.14, 95%CI [1.08; 1.19])

**Fig. 5:**
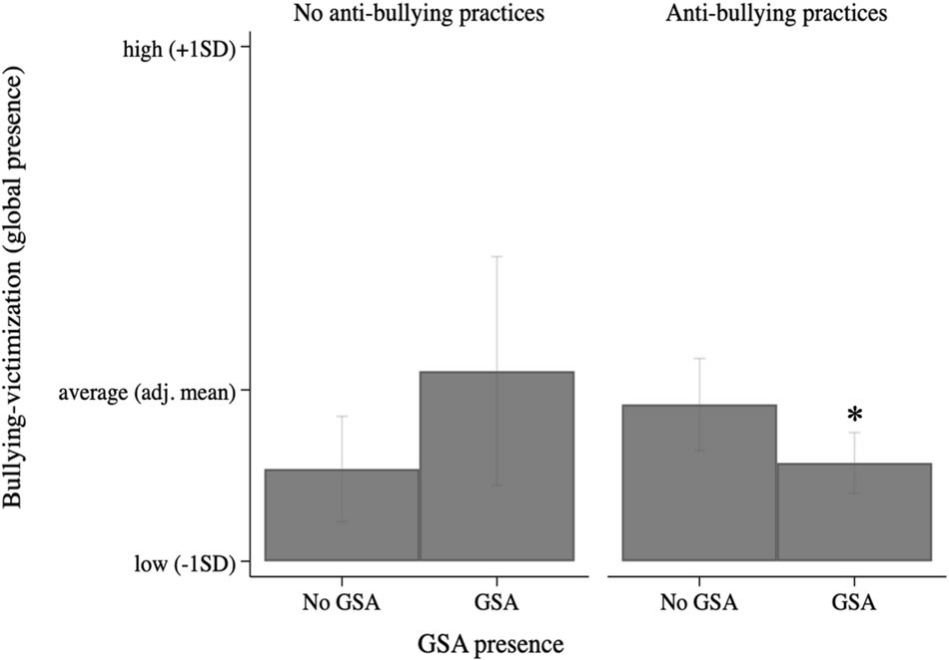
Interaction of GSA presence and anti-bullying practices on bullying-victimization (global presence). Note. *significant difference (*p* < 0.05) between no GSA versus GSA. Estimated predictive margins: for no anti-bullying practices, no GSA (Margins = 1.16, 95%CI [1.06; 1.22]) versus GSA present (Margins = 1.29, 95%CI [1.12; 1.46]; for anti-bullying practices present, no GSA (Margins = 1.24, 95%CI [1.17; 1.31]) versus GSA present (Margins = 1.15, 95%CI [1.10; 1.20])

Last, the alternative measure of bullying-victimization that disregarded frequency but focused on global presence was used. Significant interaction effects were shown for SGD-specific practices (model 3) explaining 7% of the variance, and for general anti-bullying practices (model 4) explaining 1% of the variance. No interaction effects were observed not for sexual attraction (model 2). Again, students in schools that had SGD-specific or general anti-bullying practices reported significantly less bullying-victimization when their school also had a GSA, but for those in schools without SGD-specific (Fig. 4) or general anti-bullying (Fig. 5) practices GSA presence was not associated with bullying-victimization. Thus, again, only the combination of school-led anti-bullying (and in this case, also SGD-specific) practices and GSA presence was associated with lower bullying-victimization levels.

### Sensitivity Analyses

Sensitivity analyses that included schools that were unsure whether a GSA was present at their school (compared to not having one) showed the same results as the main analyses, see Table 5. GSA presence was related to more SGD-specific social safety, in terms of sexual diversity-inclusive attitudes and safe disclosure climate, but not general social safety in terms of bullying.

**Table 5:** Linear multilevel regression with GSA unsure: social safety across combinations of GSA unsure (versus absent) and schools’ prevention activities (*N* = 8 Schools, *N* = 98 students unsure)

	Sexual diversity-inclusive attitudes				Safe disclosure climate				Bullying-victimization (frequency)				Bullying-victimization (global presence)			
	*b*	95% CI	pseudo-*R*^*2*^		*b*	95% CI	pseudo-*R*^*2*^		*b*	95% CI	pseudo-*R*^*2*^		*b*	95% CI	pseudo-*R*^*2*^	
			ind.	school			ind.	school			ind.	school			ind.	school
Main model			0.47	0.05			0.07	0.34			0.01	0.04			0.02	0.09
GSA unsure (ref = no GSA)	**0.16**	0.02; 0.27			**0.15**	0.04; 0.27			0.10	−0.20; 0.00			−0.03	−0.10; 0.03		
Sexually-diverse	**0.70**	0.53; 0.87			0.04	−0.12; 0.18			**0.33**	0.15; 0.51			**0.12**	0.09; 0.23		
SGD-specific practices	−0.01	−0.13; 0.13			0.05	−0.04; 0.18			−0.02	−0.12; 0.08			−0.03	−0.09; 0.03		
Anti-bullying practices	−0.13	−0.26; 0.01			0.05	−0.17; 0.08			0.08	−0.03; 0.18			0.04	−0.03; 0.10		

Covariates were age, gender, sexual diversity, migration background, family affluence, cohort, school-led SGD-specific practices and anti-bullying practices. Bold values represent significant effects

*Ind* individual-level, *school* school-level

## Discussion

Feeling safe and included at school is a fundamental need for adolescents, and exposure to stigma or bullying can have a relatively large impact in adolescence compared to other developmental periods (Earnshaw et al., 2022). While it is known that the presence of a Gender and Sexuality Alliance (GSA) at school can enhance social safety, this study tested for whom and in which school contexts the presence of a GSA was related to students’ social safety. Firstly, this study examined to what extent having a GSA at school was associated with both sexual diversity-specific and general social safety. Additionally, it was tested whether these associations differed 1) between sexually-diverse versus heterosexual students and 2) among students in schools with a combination of GSA and additional school practices to foster social safety, compared to those in schools that only reported to have a GSA without additional school practices. The results of multilevel regression analyses revealed that GSA presence was associated with more sexual diversity-specific safety (inclusive attitudes, safety to disclose one’s diverse sexual orientation), but solely among sexually-diverse students. Specifically, sexually-diverse students in schools with a GSA reported more inclusive attitudes about sexual diversity, and perceived their school climate as safer for disclosing one’s diverse sexual orientation. Moreover, GSA presence was linked to lower levels of general bullying-victimization, but only when the student-initiated GSA coexisted with additive school-led practices aimed at addressing bullying-victimization.

### GSA Presence and Social Safety: The Roles of Sexual Orientation and School Practices

These findings contribute to the current understanding of the role of GSA presence in social safety in several ways. Firstly, the observed relationship between GSA presence and social safety aligns with previous research conducted in other countries (e.g., Marx & Kettrey, 2016) and extends this association to the context of the Netherlands. Secondly, the findings showed that these associations between GSA presence and school safety depend on individual (i.e., sexual orientation) and school factors.

The observed link between GSA presence and more inclusive attitudes about sexual diversity among sexually-diverse students may be attributed to their frequent exposure to the positive messages and support offered by the GSA. This exposure potentially contributes to the development of a more positive perspective on same-sex attraction, empowerment (Russell et al., 2009), and may aid in reducing internalized homophobia (McCormick et al., 2015). Alternatively, it is possible that schools with sexually-diverse students who have more inclusive attitudes are more likely to have a more active GSA. Additionally, this study showed that solely sexually-diverse students reported a safer school climate for disclosing one’s diverse sexual orientation within the context of a GSA. This suggests that such safety may be personally relevant to them and may not be as pertinent to heterosexual students. Sexually-diverse students might, therefore, be more aware of the safe space that a GSA can create, facilitating more open discussions about their sexual orientation.

Moreover, the findings of the current study highlight the importance of looking at contextual embeddedness of GSAs instead of studying them as a stand-alone setting. Schools with and without a GSA only differed in general social safety—reflected by lower levels of bullying-victimization—when the student-led GSA was accompanied anti-bullying practices initiated by the school staff. This study did not explicitly test mechanisms, but it is possible that anti-bullying practices provide a supportive foundation for initiatives such as GSAs. The findings are in line with queer pedagogical theory (Lapointe, 2015) and the program theory of GSAs (Schlief et al., 2023), that emphasize that the presence of a GSA in schools should not represent isolated and marginal spaces in larger school communities, but should become ingrained in the everyday learning culture. While few, if any, empirical studies focused on the role of school practices, the findings align with studies on other contextual factors that strengthen the role of GSA presence, including inclusive school policies (Day et al., 2020) and more distal contextual factors, such as political and geographical factors that indicate available support for sexual diversity issues and resources (Calzo et al., 2020). Similarly, school-led anti-bullying practices might support a GSA through actions by school staff or school boards to tackle bullying, which creates a safer atmosphere to discuss SGD issues or to prevent negative reactions.

Last, school practices did not contribute to any associations between GSA presence and sexual-diversity specific social safety. It would, thus, be valuable to understand which other school practices or characteristics help a GSA to strengthen an inclusive climate.

### Strengths, Limitations, and Implications

Strengths of this study include the use of a large, nationally representative population sample from the Netherlands and a multilevel framework to study how both individual and school-level factors related to associations between GSA presence and four indicators of social safety. The study was, however, limited by its cross-sectional design, which made it impossible to draw conclusions about the direction of the associations; for example, whether social safety was a consequence or an antecedent of GSA presence (Baams et al., 2020). Moreover, no information was provided on which specific actions schools had taken when reporting school practices, how frequent these actions were taken (i.e., respondents may differ in their interpretation of “actively addressing” an issue), or how active the GSA was in the school, which would help to draw conclusions about “what works” in the context of a GSA. For example, it is possible that schools that reported having a GSA and actively addressing bullying (i.e., reported general anti-bullying practices) were referring to the GSA’s activities instead of anti-bullying practices beyond the GSA. In these cases, the findings of this study may be picking up on differences between being in a school with a GSA where the school attempts to be inclusive (separate from or in addition to the GSA) and being in a school with a GSA where the school is not involved in the GSA activities. Together, it would be relevant to follow up on the current findings with more detailed questions about school practices to understand how schools and students collaborate in building a safe school climate.

Further, it would have been valuable to have more comprehensive individual measures. For example, no information about gender identity/expression was provided, which would be important to also study associations among gender-diverse students, and differences compared to sexually-diverse peers—especially because the SGD-specific school practices also targeted gender diversity (Day et al., 2020). Moreover, the measures of sexual diversity-inclusive school climates were limited to sexual diversity-inclusive attitudes and safe disclosure climate, while it would be relevant to understand more in-depth how different aspects of inclusive school climates would relate to GSA presence. For example, information about experiences of sexual orientation-based victimization and safety of having same-sex relationships at school, would be informative (Kosciw et al., 2021).

Last, the final sample was too small to reliably test how combinations of individual attributes (i.e., sexual orientation) and school-led practices mutually contributed to associations between GSA presence and social safety. It would have been valuable to understand if, for example, especially for sexually-diverse students, a supporting school context strengthens the role of the GSA because it makes the school a safer space for them.

In sum, future research is needed to further understand for whom and in which schools GSAs work most effectively, by 1) testing mechanisms that account for the associations such as how school practices contribute to GSA correlates (e.g., through actions by school staff or school boards, by providing support for GSAs), 2) assessing schools’ specific actions when reporting school practices, school climate, and individual gender identity/expression, 3) using longitudinal or experimental data to test predictive effects of GSAs, and 4) using larger sample sizes to understand how individual and contextual attributes, such as sexual orientation and school practices, mutually contribute to associations between GSA presence and social safety. In such studies, it would be valuable to generalize the findings to other countries.

An important practical implication includes that it can be valuable for schools to not only rely on a GSA to improve social safety, but also on their own school practices when fostering safer school climates. Especially for adolescents, who are susceptible to peer pressure and rely on adult school staff to regulate socially challenging situations, student-initiated GSA’s alone may not fully protect students from being bullied without school staff intervention and support in the form of school-wide practices. Overall, this study exposes the need to activate school staff to change the school culture to fit the needs and desires of sexually-diverse youth, rather than provide a separate, isolated, and marginal club within an oppressive school context.

## Conclusion

While it is known that the presence of a GSA at school can enhance social safety, it is unclear for whom and in which school contexts GSA presence is linked with students’ social safety. This study showed that individual and school context play a vital role in determining the extent to which a student-led GSA is associated to social safety. GSA presence was shown to be related to more inclusive attitudes towards sexual diversity and to more perceived safety to report one’s sexual attraction, albeit solely among sexually-diverse students. Further, the combination of a GSA and general anti-bullying school practices was associated with lower levels of bullying-victimization. This implies that schools should consider both the presence of a GSA and their own policies and practices when aiming to cultivate safer and more inclusive environments. Moving forward, it is suggested that future research on GSAs should explore how they are integrated within the individual and school context, as this can provide valuable insights for creating even more impactful interventions.
